# Prevalence of Hypertension Among the Rural Adult Population in India: A Systematic Review and Meta-Analysis

**DOI:** 10.7759/cureus.69942

**Published:** 2024-09-22

**Authors:** Vijaya Kumar Uthakalla, Partha Sarathy Naidana, Raja Sekhar Yendapu, Saikiran Santosh Pissey, C U Siva Kumar Devireddi

**Affiliations:** 1 Community Medicine, Alluri Sitarama Raju Academy of Medical Sciences, Eluru, IND

**Keywords:** adults, cross-sectional studies, epidemiology, hypertension, india, meta-analysis, meta-regression, prevalence, rural, systematic review

## Abstract

Hypertension is a major cause of premature death worldwide. Studies have shown that the rural adult population in India is also experiencing this burden. To determine the overall pooled prevalence of primary hypertension in the adult rural population of India, an extensive search was conducted in various databases such as MEDLINE/PubMed, IndMED, EBSCO, CINAHL, and Google Scholar from 01/01/2014 to 16/05/2024. The studies were reviewed by two authors independently, assessing their quality and extracted data using pre-coded spreadsheets. The pooled estimates of hypertension prevalence were calculated using the Der Simonian-Laird random effects model, and subgroup, sensitivity analyses, and meta-regression were performed. In the final review, a total of 10 studies involving 30757 subjects were included. The combined pooled estimate of hypertension prevalence was 24% (95% CI: 19, 29) and there was a significant level of heterogeneity observed among the studies (I^2^=98%, Q=572.07, df=9, p<0.01). Subgroup analyses found that factors such as the year of study, region, type of BP apparatus used, sampling strategy, and BP measurement techniques had a significant impact on the prevalence of hypertension. Further analysis by meta-regression revealed that none of these covariates had a substantial influence on the prevalence (R^2^=0.21, Q=572.07, df=9, p-value<0.01). The prevalence of hypertension in adult rural populations exhibited a consistent upward trend over a period of 10 years from 2014 to 2024. Concerned policymakers should focus on the changing health needs of the rural adult population of India.

## Introduction and background

Hypertension is a leading cause of premature death worldwide [1]. As per WHO, the global target for noncommunicable diseases includes reducing the prevalence of hypertension by 33% between 2010 and 2030 [2]. Hypertension is the biggest risk factor for disease burden globally. In India, hypertension has become a prominent risk factor for mortality, with studies showing an increasing prevalence of hypertension [3]. Kearney et al. predicted that the burden of hypertension in India will almost double from 118 million in 2000 to 213.5 million by 2025 [4]. A previous systematic review reported prevalence rates of hypertension in urban and rural areas of India ranging from 13.9% to 46.3% and 4.5% to 58.8%, respectively [4]. India has seen a significant shift in disease burden from communicable to non-communicable diseases over the past two decades [5]. NCDs now account for more than 60% of total deaths, with stroke and CVD making up nearly 30% [6]. According to NFHS-5 (2019-2021), the prevalence of hypertension among women in rural India is 20.2%, and among men, it is 22.7% [7]. In India, hypertension prevalence conducted in communities over a period of three to six decades showed an increase of 30% in the urban population and 10% in the rural population [8]. The environment in which a person lives has a significant impact on their overall health, and there is a correlation between hypertension, obesity, and various modifiable socio-economic factors such as occupation, income, education, lifestyle, and living standards [9]. The prevalence of hypertension is relatively higher in urban subjects compared to rural subjects [10]. While there is considerable research on hypertension among urban adults, there is a lack of studies on the prevalence and factors associated with hypertension in rural areas. This paper aims to address this gap by exploring the current state of hypertension among rural adults in India and its implications for public health interventions and policy-making. Understanding the burden of hypertension in rural areas is crucial for developing targeted interventions and improving overall health outcomes in these communities. Conducting systematic reviews specifically focused on rural areas will provide a comprehensive understanding of the burden and impact of hypertension in these populations. This information can guide policymakers in developing interventions and programs tailored to the specific needs of rural communities. With this in mind, this study was planned to conduct systematic reviews on the prevalence of hypertension among rural adults in India from 2014 to 2024 (10 years).

## Review

Methodology

*Search Strategy for Literature* 

Two authors conducted the literature search separately. Any disagreements regarding the inclusion of studies, assessment of quality, and extraction of data were resolved by the third author. We conducted a literature search using databases such as Medline/PubMed, IndMED, CINHAL, EBSCO, and Google Scholar using both the Medical Subject Headings (Mesh) and specific keywords including “prevalence,” “epidemiology,” “hypertension,” “pulmonary arterial hypertension,” “essential hypertension,” “adults,” ‘’rural population’,’ and “India.” Boolean search operators like “AND" and “OR” were applied throughout the searching process. We conducted a literature search up until 16/05/2024. The review protocol was registered on the International Prospective Register of Systematic Reviews (PROSPERO) 2024 database under the number CRD42024541065 (Amendments: IndMED database was added to the protocol on 16/05/2024). 

Assessment of Quality and Extraction of Data 

Two authors correctly evaluated the quality of each article using Jonna Briggs Institute modified critical appraisal checklists. Two authors independently extracted the participants' characteristics age groups of participants, sex, region, prevalence, first author of the study, study place location, year of study, sampling scheme, sample size, type of BP apparatus, number of BP readings recorded, and classification cut-offs onto pre-coded spreadsheets. We extracted the data at the lowest possible level of disaggregation. We presented this review in accordance with the PRISMA statement. 

Statistical Analysis

The meta-analysis focused on the proportion of subjects classified as having hypertension. We analyzed using R and R Studio (META & METAFOR) version 4.1.1 for Forest Plot and examined heterogeneity between studies using I^2^ statistics and Cochran's Q test. We calculated all pooled estimates using the Der Simonian-Laird random effects model and reported them as a proportion with 95% CI. We visually inspected the funnel plots and set the statistical significance at a p-value less than 0.05. We conducted a meta-regression using a random intercept and a fixed slope regression analysis (maximum likelihood estimation method) and determined the regression coefficients, their 95% CI, and sensitivity analyses by excluding studies of inferior quality and those with smaller sample sizes, then implemented each strategy sequentially. 

Results

**Figure 1:**
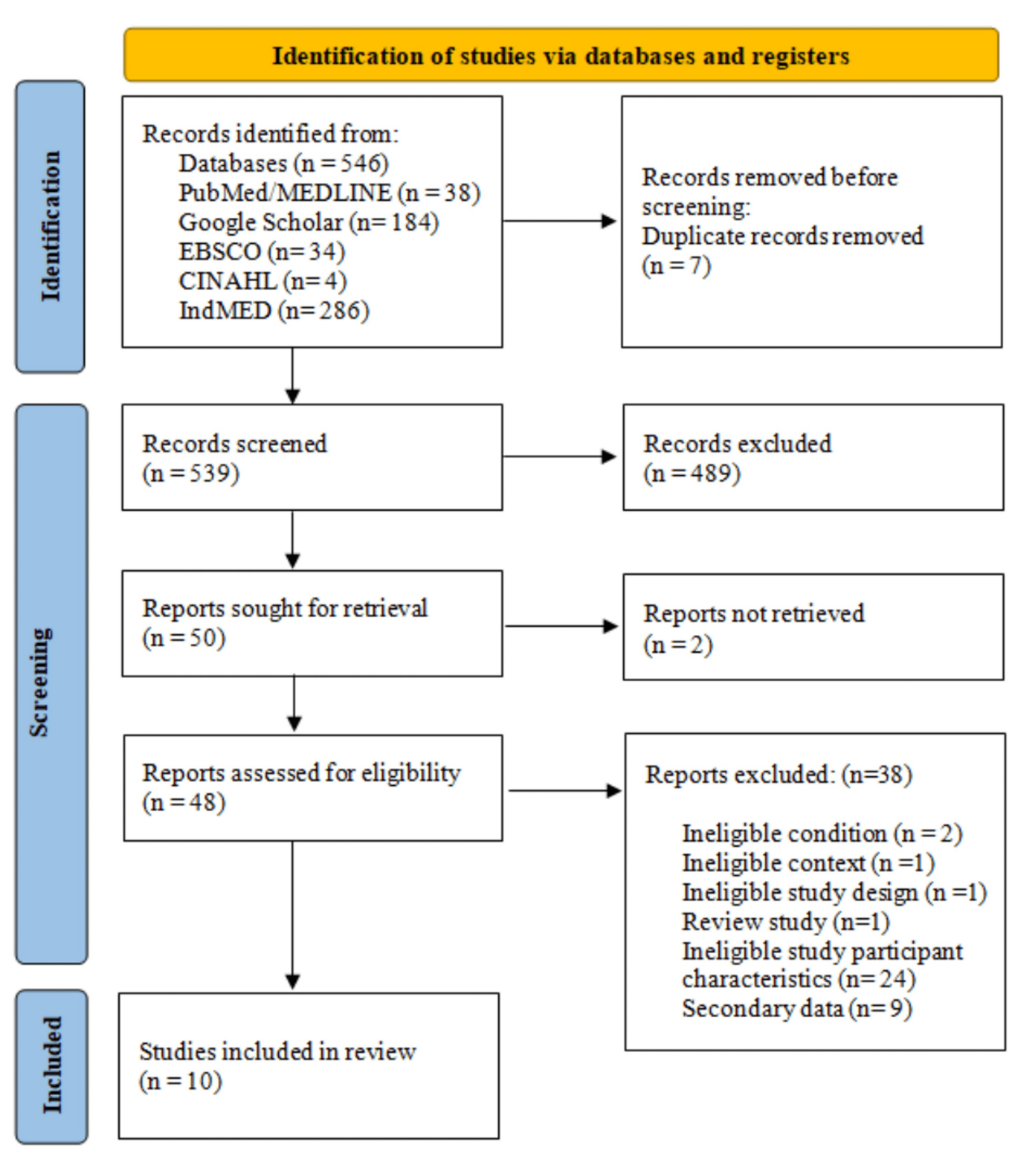
PRISMA flow chart of selection of studies for meta-analysis PRISMA, Preferred Reporting Items for Systematic Reviews and Meta-Analysis

The flow of article selection is shown in Figure 1. Finally, 10 articles were included in the review. Every study that was included was cross-sectional in nature. 30757 participants in all were enrolled in the study, of whom 10019 (5824 females and 3925 men) had hypertension. For 270 participants, there was insufficient information available about sex. The main characteristics of the study were compiled (Table 1). All other studies included a homogeneous age group (mainly above 18 to 60 years), with the exception of two that included a slightly younger age group (18-29 years and 20-30 years). Eight studies were found to be of high quality based on the quality assessment (Table 2). The study's locations were divided into five regions: north, south, west, northeast, and northwest, based on their geographic location. Studies showed a wide range of methodological variations. Most of the studies used a mercury sphygmomanometer, numerous blood pressure recordings, a random sampling technique, and a 140/90 mm Hg categorization cut-off. In a study conducted in Tamil Nadu with a sample size of 154, the prevalence of hypertension for both sexes combined (n=10 studies) ranged from as low as 7% to as high as 51% in Nagaland with 209 participants [8,9].

**Table 1:** Characteristics of included studies in the review JNC, Joint National Committee Guidelines; ING, Indian Clinical Practice Guidelines

Characteristics of included studies in the review																
Sl.no	First author	Publication year	Time period	BP apparatus	BP readings	JNC/ING	Cut-off used	Number of subjects having hypertension (prevalence of hypertension)	Age included	Age group	Gender	Place of study (state)	Region (subgroup)	Sampling Scheme	Sample size	Included
1	Boornem A et al. [8]	2024	July 2023 to August 2023	Mercury	3	JNC	≥ 140/90	11 (7%)	18 to 29 years	Not available	Not available	Tamil Nadu	SOUTH	Multistage sampling technique	154	Yes
2	Tsukru V et al. [9]	2023	Not available	Mercury	2	JNC	≥ 140/90	106 (50.7%)	18-50 years	NA	M-63 (30.1%); F-43 (20.5%)	Nagaland	NORTHEAST	Not available	209	Yes
3	Asadullah M et al. [10]	2022	2017-2019	Electronic	2	JNC	≥ 140/90	224 (26.9%)	30-60 years	Not available	Not available	Haryana State	NORTH	Multistage cluster random sampling	832	Yes
4	Basu P et al. [11]	2019	January 2017 and December 2017	Electronic	2	Indian National Guidelines	≥ 140/90	2407 (34.45%)	30-60 years	Not available	M-1013 (14.5%); F-1394 (19.95%)	Udaipur district of Rajasthan	NORTHWEST	Not available	6985	Yes
5	Gupta A et al. [12]	2016	July 2013 to December 2013	Mercury	2	JNC	≥ 140/90	310 (43.6%)	18-59 years	18-29-13 (1.8%), 30-39-53 (7.46%), 40-49-73 (10.28%), 50-59-171 (23.94%)	M-119 (16.7%); F-191 (26.9%)	Paneman galore, Karnataka	SOUTH	Stratified random sampling	710	Yes
6	Kavi A et al. [13]	2019	January 2013 to June 2015	Mercury	2	JNC	≥ 140/90	261 (26.6%)	20 and 60 years	20-29-17 (1.73%), 30-39-48 (4.89%), 40-49-88 (8.97%), >50-108 (11.02%)	M-133 (13.5%); F-128 (13.06%)	Karnataka, India	SOUTH	Simple random sampling	980	Yes
7	Kini Set al. [14]	2016	October 2011-October 2013	Mercury	2	JNC	≥ 140/90	35 (3%)	20-30 years	Not available	Not available	Karnataka, India	SOUTH	Stratified sampling	1152	Yes
8	Kumar S et al. [15]	2015	December 2008 to April 2009	Mercury	2	JNC	≥ 140/90	117 (20.38%)	18-58 years	18-27-13 (2.26%), 28-37-23 (4%), 38-47-29 (5.05%), 48-57-14 (2.43%), 58-38 (6.62%)	M-61 (10.6%); F-56(9.75%)	Nagpur	WEST	Systematic random sampling	574	Yes
9	Mallika MV et al. [16]	2015	3 months from 18-01-2010	Mercury	2	JNC	≥ 140/90	6360 (35.14%)	18-59 years	Not available	M-2398 (13.2%); F-3962 (21.9%)	Kerala, South India	SOUTH	Not available	18100	Yes
10	Zafar KS et al. [17]	2017	January 2016 to June 2016	Mercury	3	JNC	≥ 140/90	188 (17.72%)	18 to 40 years	18-29-132 (12.44%), 30-40-56 (5.27%)	M-138 (13%); F-50 (4.7%)	Uttar Pradesh, India	NORTH	Not available	1061	Yes

**Table 2:** Subgroup analysis of the pooled prevalence of hypertension among rural adults using methodological factors P-value <0.05 is statistically significant. In subgroup analysis year, region, BP guidelines, and sampling strategy are found to be statistically significant with the prevalence of hypertension among rural adults in India. BP apparatus, blood pressure measuring apparatus; BP guidelines = Joint National Committee Guidelines (JNC), Indian Clinical Practice Guidelines (ING)

Variable		Proportion	95% CI	I^2^	Cochran’s Q	p-value
All studies		24	0.19;0.29	98	572.07	<0.01
Subgroup analysis						
Year wise	2015	27	0.15;0.44	98	122.07	<0.01
	2016	14	0.01;0.78			
	2017	18	0.15;0.20			
	2019	31	0.23;0.39			
	2022	27	0.24;0.30			
	2023	51	0.44;0.58			
	2024	7	0.04;0.12			
Region	South	18	0.10;0.29	98	83.72	<0.01
	West	20	0.17;0.24			
	North	22	0.14;0.32			
	Northeast	51	0.44;0.58			
	Northwest	34	0.33;0.36			
BP apparatus	Mercury	21	0.15;0.30	98	2.84	0.09
	Electronic	31	0.24;0.39			
BP guidelines	JNC	22	0.16;0.30	98	9.22	<0.01
	ING	34	0.33;0.36			
Sampling strategy	Random	17	0.10;0.29	98	5.76	0.02
	Not available	33	0.28;0.38			
No. of BP readings	2	27	0.22;0.32	98	3.63	0.06
	3	12	0.05;0.27			

Random Effects Pooled Estimate of the Prevalence of Hypertension

**Figure 2:**
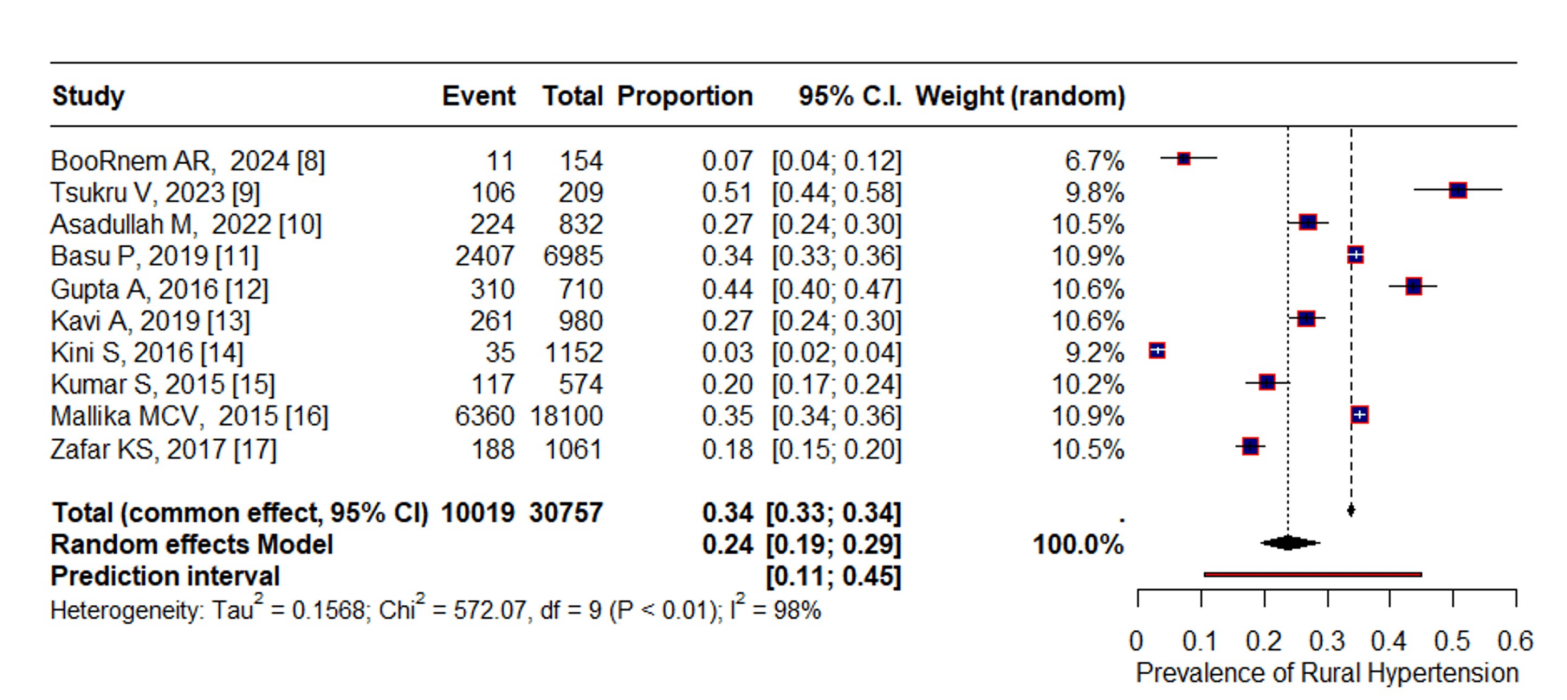
Random effects pooled estimate of the prevalence of hypertension among the rural adult population of India The studies found that the pooled prevalence of hypertension among adults in rural India showed statistically significant heterogeneity (p<0.01).

The prevalence of hypertension in the adult rural population of India was estimated to be 24% (95% CI: 19% to 29%) (Figure 2). Significant heterogeneity was observed among the studies (I^2^=98% and Cochran's Q=572.07, df=9, p<0.001). The pooled estimate of hypertension in males was 32% (95% CI; 24.9% to 40%), while in females it was 27% (95% CI; 22.4% to 32.1%).

Subgroup Analyses

We included all studies in subgroup and sensitivity analyses. From 2014 to 2024, the prevalence of hypertension was 24% (27% to 7%). This increase was statistically significant (p<0.05). The region-wise prevalence of hypertension from south to northwest (18% to 34%) was also found to be statistically significant (p<0.05). The type of blood pressure apparatus and the prevalence of hypertension between mercury and electronic sphygmomanometers (21% vs. 31%) was not statistically significant (p>0.05). The blood pressure measurement guidelines that used Joint National Committee Guidelines (JNC) and those studies that used Indian Clinical Practice Guidelines (ING) (22% vs. 34%) had a significant statistical association with the prevalence of hypertension (p<0.05). The studies that used random sampling strategy and non-random sampling strategy (17% vs. 33%) were found to be significantly associated with hypertension (p<0.05). The studies that used two blood pressure readings and three blood pressure readings (27% to 12%) were not associated with hypertension (p>0.05) (Table 3).

**Table 3:** Quality assessment of studies by JBI critical appraisal method included in the review A smaller sample size study was Boornema AR, 2024 [8]. Low-quality study was Tsukuru V, 2023 [9].

Citation	Q1	Q2	Q3	Q4	Q5	Q6	Q7	Q8	Q9	Total score	Percentage
Boornema AR, 2024 [8]	2	2	2	2	2	2	2	2	2	18	100%
Tsukuru V, 2023 [9]	2	0	0	2	2	2	2	2	2	14	77.77%
Asadullah M, 2022 [10]	2	2	2	2	2	2	2	2	2	18	100%
Basu P, 2019 [11]	2	0	1	2	2	2	2	2	2	15	83.33%
Gupta A, 2016 [12]	2	2	2	2	2	2	2	2	2	18	100%
Kavi A, 2019 [13]	2	2	2	2	2	2	2	2	2	18	100%
Kini S, 2016 [14]	2	2	2	2	2	2	2	2	2	18	100%
Kumar S, 2015 [15]	2	2	2	2	2	2	2	2	2	18	100%
Mallika MCV, 2015 [16]	2	0	1	2	2	2	2	2	2	15	83.33%
Zafar KS, 2017 [17]	2	0	1	2	2	2	2	2	2	15	83.33%
%	100	60	60	100	100	100	100	100	100		

Meta-Regression Analysis

The covariates that had a p-value <0.20 in the subgroup analysis were included in a random effects meta-regression analysis. However, none of the covariates were found to be statistically significant. The overall model, which had an R^2^=0.21, was also not significant with a p-value >0.01 (Table 4).

**Table 4:** Random effects meta-regression analysis, the effect of covariates on the prevalence of hypertension R^2^=21%, I^2^=98.68%, Q=394.64, df=5, p< 0.001.

Variable	Estimate	SE	Z-values	P-value	95% CI	
Region	0.2684	0.8921	0.3009	0.7635	1.4801	2.0169
Year	0.0838	0.7834	0.1070	0.9148	1.4516	1.6192
BP guidelines	0.0045	1.1302	0.0040	0.9968	2.2106	2.2196
Sampling strategy	0.7401	0.7630	0.9699	0.9699	0.7554	2.2356
Intercept	2.7528	1.6096	1.7102	0.0872	5.9076	0.4020

Sensitivity Analyses

**Figure 3:**
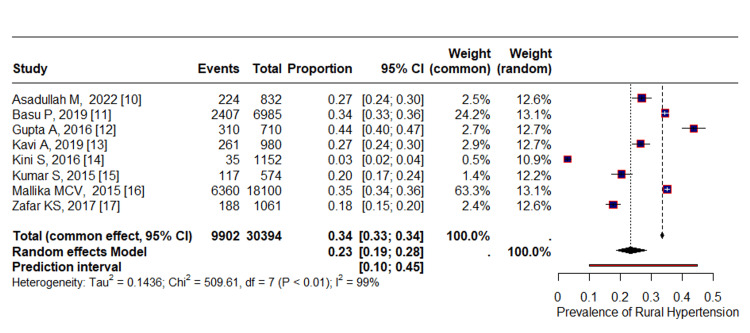
Forest plot of pooled estimate after removing both low-quality and less sample size studies After removing Boornema AR and Tsukru [8,9]. The heterogeneity among the studies was found to be statistically significant (p-value <0.01).

During our analysis, we performed a sensitivity analysis by systematically excluding individual studies to identify any factors contributing to heterogeneity. The results showed that the overall pooled estimates did not vary significantly when each study was removed. However, when we excluded a low-quality study, the pooled estimate slightly decreased from 24% to 22%. Conversely, when we excluded a study with less sample size, the pooled estimate slightly increased from 24% to 26% and when both low-quality and less sample size studies were removed then the pooled estimate was 23% (Table 5, Figure 3).

**Table 5:** Pooled prevalence of hypertension among rural adults after sensitivity analyses After removing low-quality and small sample size studies, the sensitivity analysis found statistically significant heterogeneity (p<0.01) in relation to the prevalence of hypertension among rural adults in India.

Variable	Proportion	95% CI	I^2^	Cochran’s Q	P-value
Removal of low-quality study	22	0.17;0.26	99	545.86	<0.01
Removal of less sample size study	26	0.21;0.31	99	535.60	<0.01
Removal of both low quality and less sample size studies	23	0.19;0.28	99	509.61	<0.01

Publication Bias

**Figure 4:**
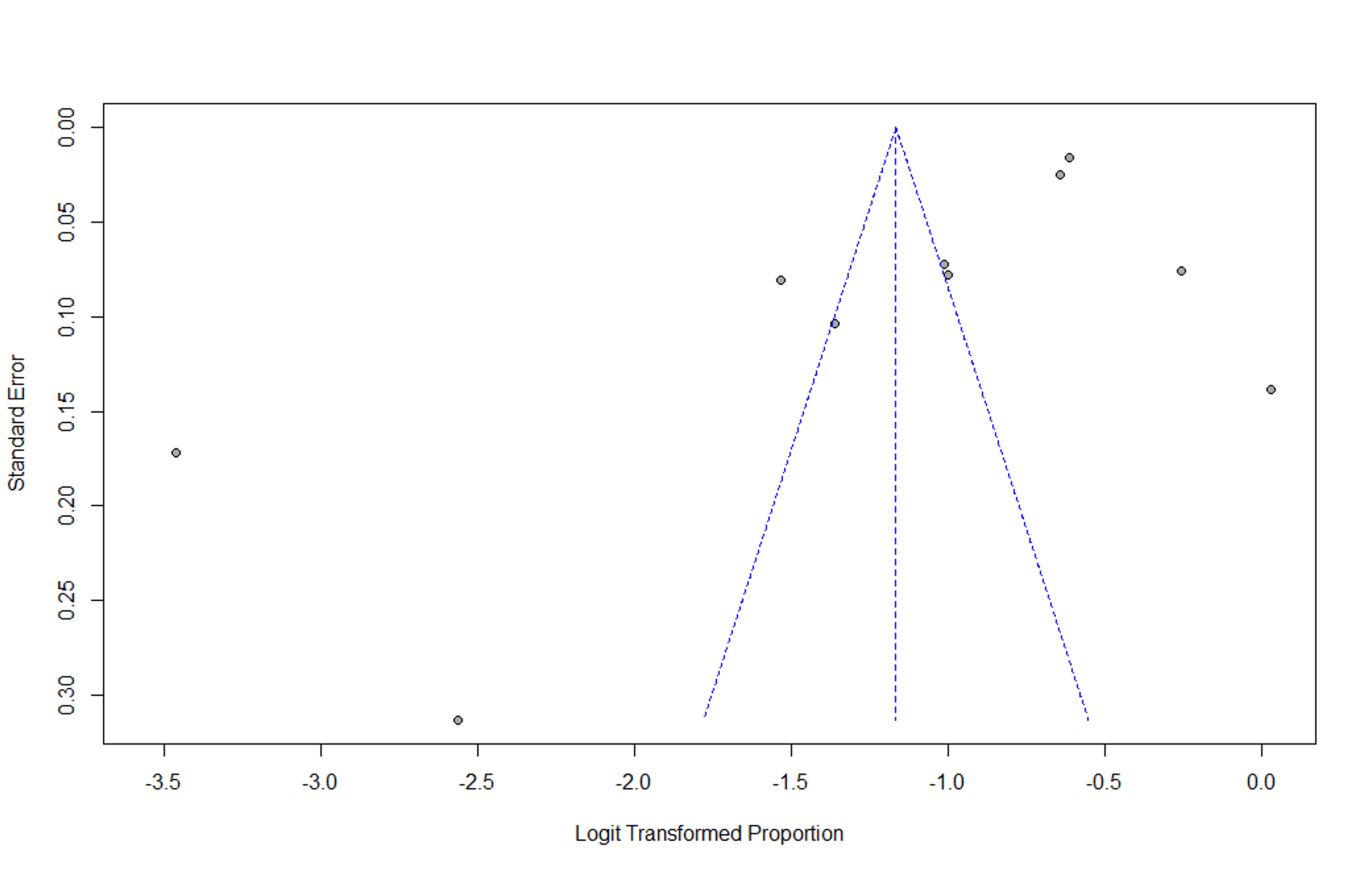
Funnel plot of prevalence of hypertension among rural adults in India The funnel plot indicated slight asymmetry from Egger's test; there was no clear evidence of publication bias with a p-value of 0.063.

The publication bias does not have a substantial impact on a meta-analysis of prevalence studies, we found several smaller studies with significant effects, which we found out in our sensitivity analysis. Additionally, the funnel plot indicated slight asymmetry, but with a p-value of 0.063 from Egger's test; there was no clear evidence of publication bias or any bias that may have gone unnoticed (Figure 4).

Discussion

The findings of this study, which included a systematic review and meta-analysis of data from 10 studies involving a total of 30757 participants, revealed that out of the 10019 individuals with hypertension, there were 5824 females and 3925 males. The overall pooled prevalence of hypertension among the rural adult population in India was found to be 24% (19% to 29%). The studies had significant heterogeneity between them, which can be explored by subgroup analysis.

In a study conducted by Anchala R et al. in 2014, they reported a hypertension prevalence of 27.6% (95% CI; 23.2% to 32.0%) among rural adults in India, which was higher than the pooled prevalence of 24% found in our study. The discrepancy in prevalence estimates could be attributed to the inclusion of individuals above the age of 60 in their study [18].

Another study done by Midha T et al., 2000-2012 years, reported the prevalence of hypertension as 17.9% (95% CI;17.5%-18.3%) among rural adults in India, which was lower than our study (24%) and shows a significant increase in the prevalence of hypertension from 17.9% to 24% from 2000 to 2024 [19].

The study done by Bansal SK in Uttarakhand villages in 2010 reported a higher prevalence of hypertension of 32.3% (95% CI; 23.8% to 30.9%), which might be due to the inclusion of more than 15 years and ≥60 years of age group in their study [20].

Another study conducted by Yun Hang et al., from 2000 to 2018, reported a prevalence of hypertension of 24.4% (95% CI; 23.8-30.9), which corresponds similarly to our results of 24% among rural adults in India [21].

Another study conducted by Chowdary et al. reported a low prevalence of 15% (95% CI; 13% to 16%); this variation may be due to the fact that the majority of the studies included were ≥35years (13 studies), ≥25 years (11 studies) and ≥18 years (8 studies) and reported low prevalence compared to our study (24%) [22].

Systematic review and meta-analysis done by Nasir Sani (2000-2021), West Africa, and Bao et al., China (1959-2018), reported a higher prevalence of hypertension of 27.4% and 26% than our study (24%), which might be due to inclusion of more than 60 years age group and variation in socio-demographic, genetic, and cultural practices between West Africa, China, and India [23,24].

In our study, the pooled estimate of hypertension was higher among males and age group >30 years; similar results were reported by the published studies [11-17,25,26].

The univariate analysis revealed significant associations between methodological factors and the prevalence of hypertension. The factors included were region, blood pressure classification, year, and sampling strategy, and none of them influenced prevalence when the meta-regression model was applied (R^2^=0.21%).

Strengths and limitations

The main strength was the standard search strategy followed, Jonna Briggs institute critical appraisal done, and explored heterogeneity by subgroup analysis. The inclusion of a large sample size allowed us to obtain an accurate and precise estimate of the pooled prevalence of hypertension. Several covariates were associated with pooled estimates in univariate analysis and applied meta-regression for the influence of the covariates on hypertension. No evidence of publication bias.

Two studies did not report male and female populations in the study; different sampling strategy methods were used, which caused errors in estimation; calibration of the BP apparatus was not reported, and a smaller number of studies were included in the review (10 studies).

Future research directions/implications

To enhance future research on the rural adult population, it is crucial to study issues like detailed lifestyle factors, dietary patterns, and personal habits of the rural adult population, majority of them were adopting urban lifestyles due to rapid urbanization and industrialization.

## Conclusions

The prevalence of hypertension among rural adults in India is a pressing public health issue that requires targeted interventions and policies. By addressing common risk factors and improving awareness and access to healthcare services, healthcare professionals can work toward reducing the burden of hypertension in rural populations. Continued research and collaborative efforts are essential to effectively tackle this growing health challenge in rural India.
